# Dynamic contrast-enhanced MR and PET/CT findings of uterine sarcomatoid carcinoma: a case report

**DOI:** 10.1186/s12905-020-01084-5

**Published:** 2020-10-06

**Authors:** Tingting Cui, Yanfang Jin, Bin Li, Jiyuan Li, Yunlong Yue

**Affiliations:** Department of MR, Beijing Shijitan Hospital, Capital Medical University, Beijing, China

**Keywords:** Uterus, Sarcomatoid carcinoma, MRI, PET-CT

## Abstract

**Background:**

Sarcomatoid carcinoma (SC) is a malignant tumour composed of spindle cells. The incidence of SC is low, especially in the uterus. The imaging features of uterine sarcomatoid carcinoma (USC) are rarely reported. We report a case of USC and discuss the dynamic contrast-enhanced MR (DCE-MR) and PET/CT findings.

**Case presentation:**

A 69-year-old woman presented to the Department of Gynaecology with vaginal bleeding. Ultrasound examination discovered a heterogeneous mass in the cervix. Then, MRI examination of the pelvis was performed. On T2-weighted images, the uterus was replaced by an ill defined and diffuse lesion with inhomogeneous intensity. On T1-weighted images, the lesion appeared with signal hypointensity and was heterogeneously enhanced with contrast material. Additionally, enlarged lymph nodes were found in the pelvic cavity. PET/CT demonstrated high uptake in the region of the uterus and pelvic lymph nodes, which was consistent with MRI findings. The radiologists diagnosed the patient with malignant uterine lesions. The patient underwent hysterectomy and bilateral adnexectomy with pelvic lymph node dissection. Then, systemic radiotherapy and chemotherapy were performed. USC with lymph node metastasis was diagnosed with the help of immuno-histochemical analysis. There was no treatment related complication and no evidence of tumour recurrence at the postoperative 6-month follow-up.

**Conclusion:**

MRI and PET/CT features are sufficient to indicate the malignant nature of a USC, but they are not pathognomonic.

## Background

SC is a rare spindle cell carcinoma [1, 2]. It is most commonly found in the lung, followed by the liver, kidney, bladder and gastrointestinal tract [3, 4]. Clinically, SC is an aggressive malignancy with a high rate of metastasis. It responds poorly to treatment and has a poor prognosis (5-year survival rate of 15–42%) [1, 5].

To date, 18 cases of sarcomatoid squamous cell carcinoma of the cervix have been described in the English-language literature. However, only two reports have discussed the MRI or PET features of USC. Here, we present a case of pathologically confirmed USC and the accompanying DCE-MRI and PET/CT findings.

## Case presentation

A 69-year-old woman with a past medical history of hypertension, tuberculosis, and meningioma presented with abnormal vaginal bleeding for 10 days as well as fever. Furthermore, her family history was unremarkable. Physical examination revealed a barrel-shaped uterus. The patient felt mild tenderness upon palpation. Laboratory studies noted an elevated carbohydrate antigen 125 (CA125) level of 201.1 U/ml, while the carcinoembryonic antigen (CEA) and carbohydrate antigen 19–9 (CA19–9) levels were normal.

**Fig. 1:**
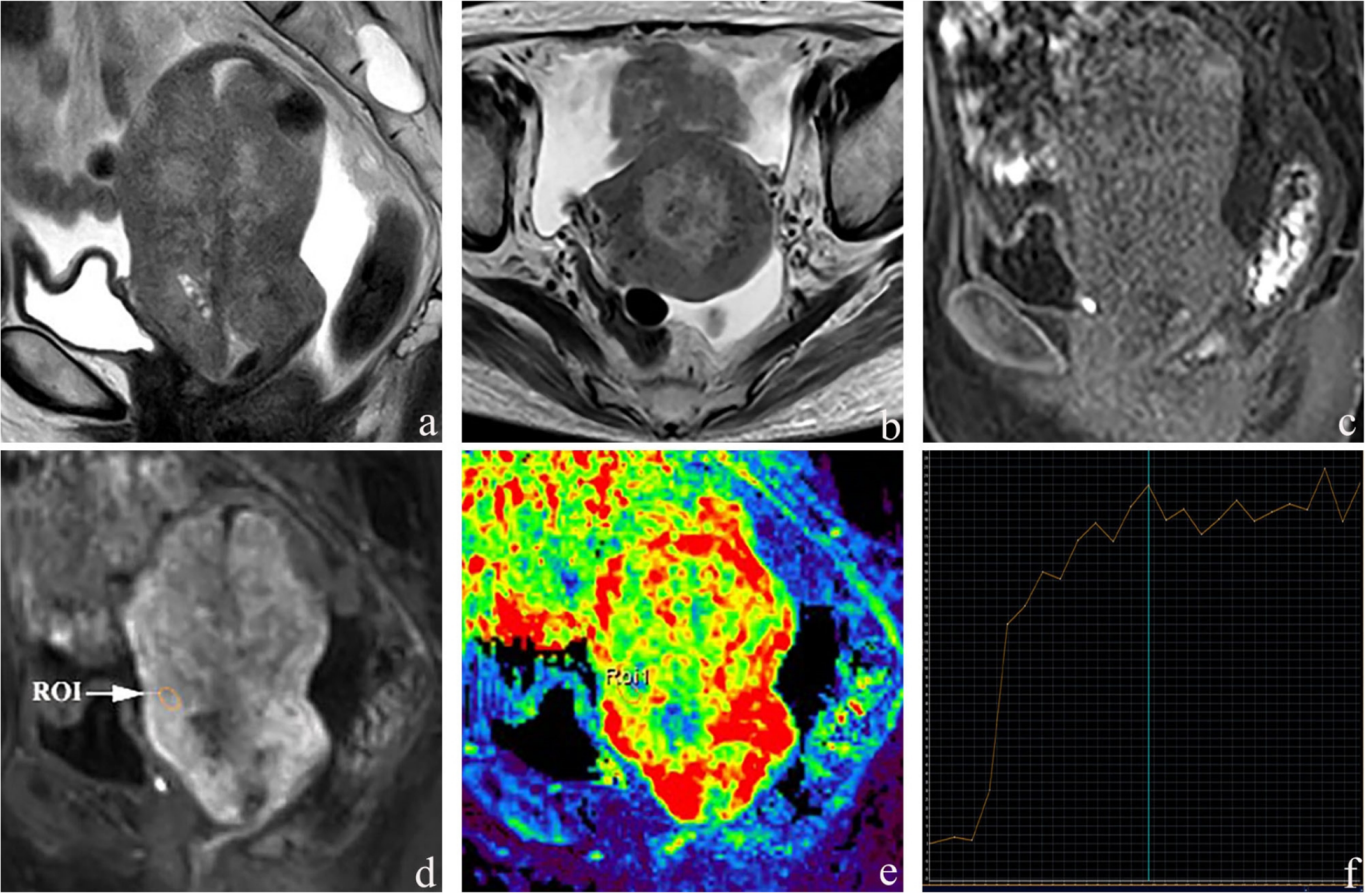
MRI images. **a** Sagittal T2-weighted image shows that the uterus was replaced by diffuse inhomogeneous signal intensity. **b** Axial T2-weighted image shows that the lesion is ill defined and diffusely invades the myometrium. **c**, **d** Sagittal T1-weighted image shows the lesion appeared with hypointensity and inhomogeneous enhancement. **e** Colour perfusion map shows the slightly lower vascularity of the tumour (green area), while the greater vascularity is depicted in red (the normal myometrium). **f** The dynamic enhanced curve of the USC: avid enhancement, with both early and persistent enhancement

**Fig. 2:**
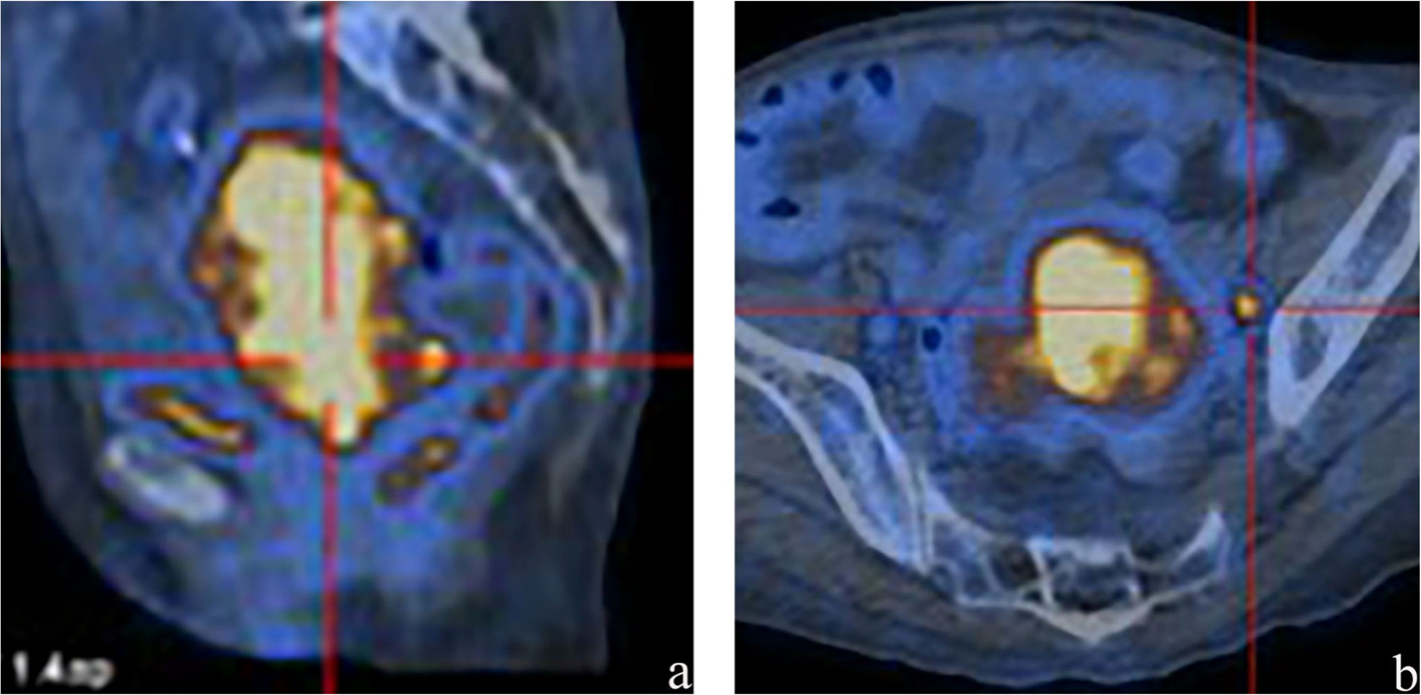
PET/CT images. **a** Sagittal PET/CT fusion image shows abnormal FDG accumulation in the uterus, and the SUVmax is 24.8. **b** Axial PET/CT fusion image shows abnormal FDG accumulation in the pelvic lymph nodes, and the SUVmax is 19.3

The patient underwent a pelvic Two-dimensional ultrasound that showed an enlarged uterus and a heterogeneous mass in the cervix. Then, MRI examination of the pelvis was performed with a 1.5 Tesla MR scanner (Ingenia 1.5 T; Philips Healthcare, The Netherlands). The T2-weighted images showed that the uterus was replaced by an ill defined and diffuse lesion with inhomogeneous intensity. On T1-weighted images, the lesion appeared with hypointensity. The lesion showed invasive growth with enlarged lymph nodes in the pelvic cavity. DCE-MRI demonstrated inhomogeneous enhancement and resulted in a plateaued curve with a prolonged scanning time (Fig. 1). PET/CT fusion images showed abnormal FDG accumulation, with maximum SUVs of 24.8 and 19.3 for the uterus and pelvic lymph node, respectively (Fig. 2). Therefore, uterine malignancy with nodal metastasis was diagnosed.

**Fig. 3:**
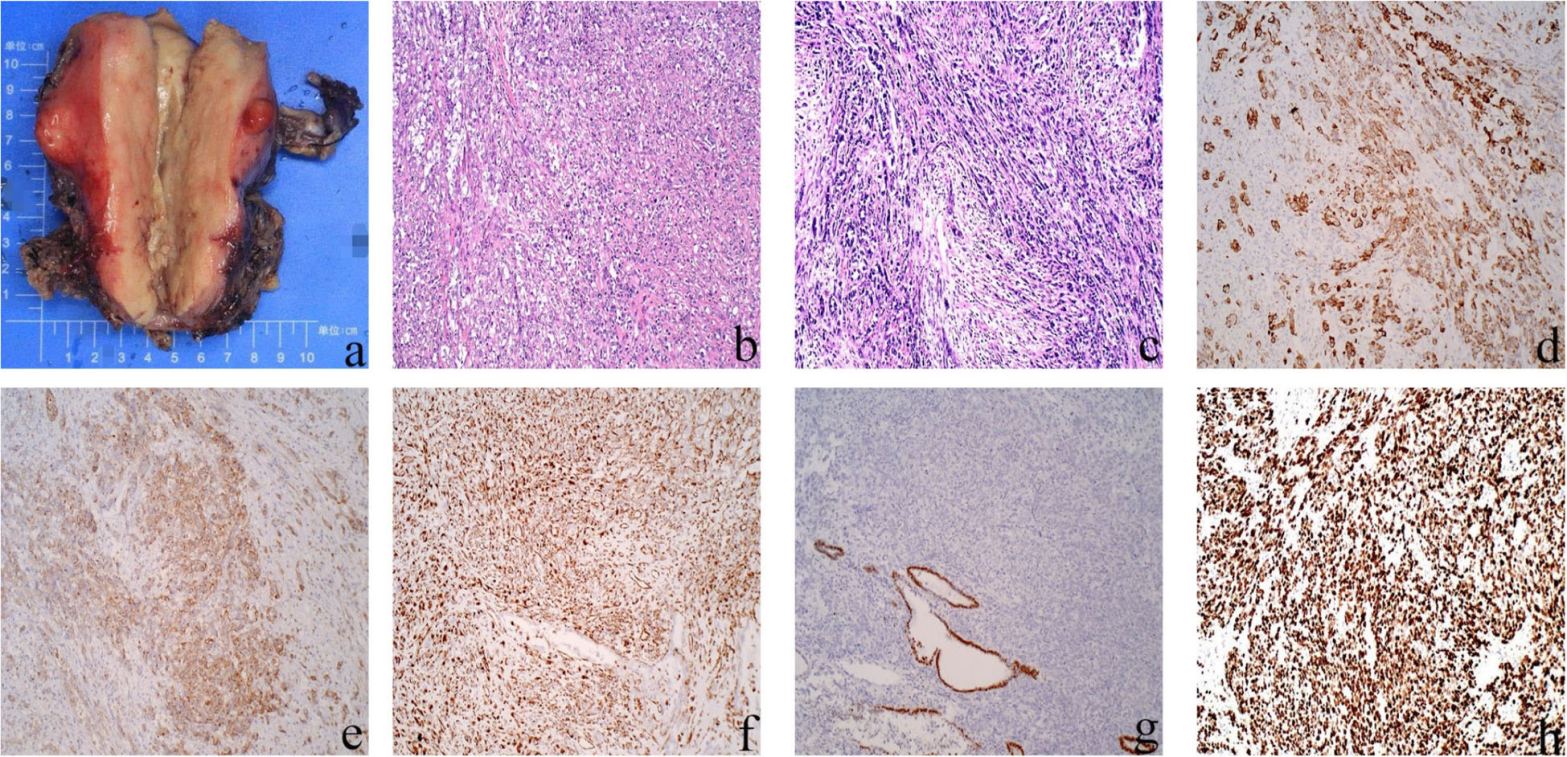
Gross surgical specimen and photomicrographs. **a** The enlarged uterus with thickened myometrium. **b** Spindle cells in the cervix (HE, × 100). **c** Spindle cells in the corpus (HE, × 100). **d** Cytoplasmic positive immunoreactivity for CK (original magnification × 100). **e** Cytoplasmic positive immunoreactivity for EMA (original magnification × 100). **f** Cytoplasmic positive immunoreactivity for vimentin (original magnification × 100). **g** Cytoplasmic negative immunoreactivity for PAX-8 (original magnification × 100). **h** Ki-67 index is 90%

The patient underwent hysterectomy and bilateral adnexectomy with pelvic lymph node dissection. Macroscopically, the uterus was enlarged with thickened myometrium, which was grey and yellow on the cut surface with poor elasticity and brittle texture. Additionally, some nodules could be seen in the myometrium. Histologically, haematoxylin and eosin (HE) stained sections showed that the uterine tumour was composed of spindle cells and nodal metastasis was confirmed. Immunohistochemical analysis supported the diagnosis of USC (Fig. 3). The patient received postoperative systemic radiotherapy and chemotherapy. The patient remained well and has no evidence of recurrence at the postoperative 6-month follow-up.

## Discussion and conclusions

SC is an extremely rare malignant tumour of the uterus that has not been listed in the WHO classification as a separate entity. Because of the rarity SC, its differential diagnosis often includes aggressive uterine lesions. As an entity, USC should not be confused with uterine carcinosarcoma. Pathologically, USC is characterized by the presence of spindle cells, showing broad-spectrum epithelial and mesenchymal markers. In contrast to uterine carcinosarcoma, USC does not express malignant heterologous elements, such as chondrosarcoma or osteosarcoma [6–10].

To the best of our knowledge, only two reports have discussed the MRI or PET features of USC in the literature [11, 12]. Shrivastava et al. found that USC showed hyperintensity on T2WI and isointensity on T1WI similar to cervical mass lesions with extra-uterine extension [11]. Milind et al. described abnormal FDG accumulation of USC in the region of the cervix and the upper vagina by PET, with maximum SUVs of 5.7 and 10.6 for the vagina and the cervix, respectively [12]. In our case, DCE-MRI showed that USC presents with inhomogeneous signal intensity on T2WI and heterogeneous enhancement on T1WI, as well as cystic degeneration and necrosis, and the lesion was diffuse rather than a well-defined mass. PET/CT images showed abnormal FDG accumulation, with maximum SUVs of 24.8 and 19.3 for the uterus and pelvic lymph node, respectively. Our MRI and PET/CT findings of USC were different from previous reports and therefore may not be specific for USC.

Distinguishing USC from uterine sarcoma is not possible because these tumours share many imaging characteristics, including heterogeneity, cystic degeneration and necrosis [13–15]. Therefore, the final diagnosis must rely on immunohistochemistry. Although a precise diagnosis of USC could not be established preoperatively, the combination of clinical, MRI and PET/CT findings support the diagnosis of a malignant disease. The patient was operated by doctor with proper expertise followed by adjuvant radiotherapy and chemotherapy. The patient remained well and has no evidence of recurrence at the postoperative 6-month follow-up.

USC is a rare and aggressive tumour, and its imaging features may not be pathognomonic. The recognition of features that are suggestive of a malignant condition is sufficient to assist the surgeon in managing such patients.

## Data Availability

The datasets used during the current study are available from the corresponding author on reasonable request.

## Abbreviations

SC: Sarcomatoid carcinoma
USC: Uterine sarcomatoid carcinoma
DCE-MRI: Dynamic contrast-enhanced MR
CA125: Carbohydrate antigen 125
CEA: Carcinoembryonic antigen
CA19–9: Carbohydrate antigen 19–9
HE: Hematoxylin and eosin
